# N-acetylcysteine Reduces Inflammasome Activation Induced by SARS-CoV-2 Proteins In Vitro

**DOI:** 10.3390/ijms232314518

**Published:** 2022-11-22

**Authors:** Javier Milara, Fernando Martínez-Expósito, Paula Montero, Inés Roger, Maria Amparo Bayarri, Pilar Ribera, Miriam Natsuki Oishi-Konari, Jose Ramón Alba-García, Enrique Zapater, Julio Cortijo

**Affiliations:** 1 Department of Pharmacology, Faculty of Medicine, University of Valencia, 46014 Valencia, Spain; 2 Pharmacy Unit, University General Hospital Consortium, 46014 Valencia, Spain; 3 Centro de Investigación Biomédica en Red de Enfermedades Respiratorias (CIBERES), Health Institute Carlos III, 46014 Valencia, Spain; 4 ENT Department, Consorci Hospital General Universitari de Valencia, 46014 Valencia, Spain; 5 Faculty of Health Sciences, Universidad Europea de Valencia, 46010 Valencia, Spain; 6 Research and Teaching Unit, University General Hospital Consortium, 46014 Valencia, Spain

**Keywords:** inflammasome, COVID-19, SARS-CoV-2, N-acetylcysteine

## Abstract

Inflammasome activation is one of the first steps in initiating innate immune responses. In this work, we studied the activation of inflammasomes in the airways of critically ill COVID-19 patients and the effects of N-acetylcysteine (NAC) on inflammasomes. Tracheal biopsies were obtained from critically ill patients without COVID-19 and no respiratory disease (control, *n* = 32), SARS-CoV-2 B.1 variant (*n* = 31), and B.1.1.7 VOC alpha variant (*n* = 20) patients. Gene expression and protein expression were measured by RT-qPCR and immunohistochemistry. Macrophages and bronchial epithelial cells were stimulated with different S, E, M, and N SARS-CoV-2 recombinant proteins in the presence or absence of NAC. NLRP3 inflammasome complex was over-expressed and activated in the COVID-19 B.1.1.7 VOC variant and associated with systemic inflammation and 28-day mortality. TLR2/MyD88 and redox NOX4/Nrf2 ratio were also over-expressed in the COVID-19 B.1.1.7 VOC variant. The combination of S-E-M SARS-CoV-2 recombinant proteins increased cytokine release in macrophages and bronchial epithelial cells through the activation of TLR2. NAC inhibited SARS-CoV-2 mosaic (S-E-M)-induced cytokine release and inflammasome activation. In summary, inflammasome is over-activated in severe COVID-19 and increased in B.1.1.7 VOC variant. In addition, NAC can reduce inflammasome activation induced by SARS-CoV-2 in vitro, which may be of potential translational value in COVID-19 patients.

## 1. Introduction

The COVID-19 pandemic, caused by severe acute respiratory syndrome coronavirus 2 (SARS-CoV-2), has caused the death of more than 6.3 million people worldwide and remains uncontrolled. The virus primarily infects the respiratory tract, causing fever, dry cough, anosmia, and dyspnoea, as the common symptoms, but as many as 10–15% of patients can develop severe pneumonia, with can progress to hypoxia and acute respiratory distress syndrome (ARDS), requiring mechanical ventilation with a poor prognosis [1,2]. 

Severe COVID-19 is commonly treated with supportive care, and only a few treatments have been approved with a limited reduction in mortality [3,4,5,6,7,8]. Vaccines against SARS-CoV-2 are the main preventive strategy to control the pandemic, but the administration of vaccines worldwide is limited. Furthermore, the appearance of new immune-evasive viral variants and the probability of reaching immediate immunity justify the need for additional treatments to mitigate disease progression. 

The inappropriate hyperinflammatory response is recognized as the cause of many severe cases of COVID-19, driven by exaggerated inflammatory cytokine release [9]. Since the beginning of the pandemic, it has been proposed that the over-activation of the inflammasome could participate in the hyperinflammatory response of SARS-CoV-2. A role for inflammasome-driven hyperinflammation in COVID-19 pathophysiology in patients is now recognized, but only a few months ago has been demonstrated that inflammasome activation in infected macrophages can drive COVID-19 pathology in animal models [10,11,12].

Typically, inflammasomes are multiprotein cytosolic complexes in which canonical sensors, such as NLRP3, oligomerize and recruit the adaptor apoptosis-associated speck-protein containing a CARD (ASC) to form inflammasome specks, within which the inflammatory caspase 1 is recruited and activated. Upon activation, caspase 1 processes pro-IL-1β and pro-ΙL-18 into their functional cytokine forms, which initiate the production of several other cytokines such as IL-6, IL-8, and TNFα, all of them overexpressed in severe COVID-19 [13,14]. The activation of NLRP3 inflammasome can be triggered by a broad number of pathogen-associated molecular patterns (PAMPs) or damage-associated molecular patterns (DAMPs) such as cytosolic K^+^ efflux, Ca2^+^ cytosolic influx, the release of reactive oxygen species (ROS), or ATP from damaged cells [15] between others. 

SARS-CoV-2 can mediate innate immune stimulation and inflammation via toll-like receptor (TLR) 2 activation, promoting MyD88 and downstream nuclear factor (NF)-κB, mitogen-activated protein kinases (MAPKs) and interferon (IFN) regulatory factors (IRFs) activation which can trigger the expression of several pro-inflammatory cytokines, innate immune sensors, such as NLRP3, and production of IFNs [16]. 

Recent findings indicate the potential role of hydrogen sulfide (H2S) as a fundamental host defense factor against SARS-CoV-2 infection [17], showing that low serum levels of H2S inversely correlate with inflammatory biomarkers such as IL-6 and C-reactive protein (CRP), and also associated with a poor prognosis in severe COVID-19 patients [18]. In addition, reduced glutathione (GSH) may underlie the serious manifestations and death from COVID-19 [19]. N-acetylcysteine (NAC) can enhance the endogenous production of H2S and replenish intracellular reduced GSH pools. In addition, NAC has shown the ability to restore the intracellular redox imbalance as well as to reduce different inflammatory pathways mediated by Nf-κB, suggesting antioxidant, anti-inflammatory, and antiviral properties [20,21,22]. To date, the available data on the efficacy of NAC therapy in COVID-19 is scarce and controversial [23,24,25,26,27]. 

In this work, we characterized the inflammasome activation in the respiratory tract of critically ill patients with and without COVID-19. In addition, we explored the effect of NAC on inflammasome activation and inflammation induced by SARS-CoV-2 in vitro. Results provided in this work showed that (1) the inflammasome proteins are particularly overexpressed in critically ill patients with COVID-19, (2) SARS-CoV-2 enhances inflammasome proteins and inflammation through TLR2 activation, (3) NAC is able to inhibit inflammasome activation in cells stimulated with SARS-CoV-2 proteins through the inhibition of Nf-κB and NLRP3. The results provided in this work may be of potential value to understanding the effects of NAC in COVID-19 patients.

## 2. Results

### 2.1. Inflammasome Components Are Overexpressed in Airway Tissue of COVID-19 Critically Ill Patients

Tracheal tissue segments were obtained to study inflammasome activation in airways from critically ill COVID-19 patients of the Spanish first wave (B.1 variant; *n* = 31 patients) and third wave (B.1.1.7 VOC alpha variant; *n* = 20) and compared with critically ill non-infected patients (*n* = 32). Clinical characteristics are described in Table 1.

**Figure 1:**
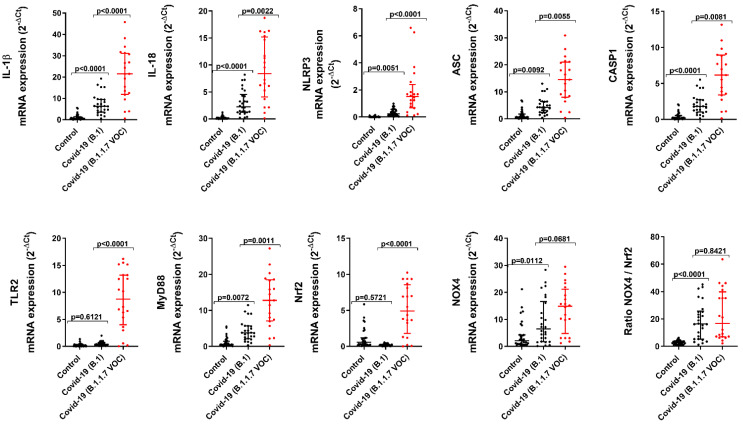
Inflammasome, innate immune and redox element gene expression in critically ill COVID-19 tracheal biopsies. Tracheal biopsies were obtained from invasive ventilated critically ill patients without COVID-19 and no respiratory disease/infections (control, *n* = 32), SARS-CoV-2 B.1 variant (*n* = 31), and B.1.1.7 VOC alpha variant (*n* = 20). Total mRNA was extracted and quantified by RT-qPCR to analyze the expression of inflammasome genes IL-1β, IL-18, NLRP3, ASC, CASP1, innate immune genes TLR2, MyD88, and redox Nrf2 and NOX4 genes. Data are presented as scatter dot blots with median and interquartile range values. *p*-values are based on the Kruskal-Wallis test and Dunn’s post hoc test for multiple comparisons.

The mRNA expression of the products of the inflammasome activation, such as IL-1β and IL-18, was overexpressed in COVID-19/B.1 patients (Figure 1; *p* < 0.0001 vs. control patients) and overexpressed in COVID-19/B.1.1.7 (Figure 1; *p* < 0.0001 vs. COVID-19/B.1 patients). Accordingly, the expression of the inflammasome components such as NLRP3, ASC, and caspase 1 was also overexpressed in COVID-19/B.1 patients and overexpressed in COVID-19/B.1.1.7 variant (Figure 1; *p* < 0.01 vs. COVID-19/B.1 patients) suggesting a more robust activation of inflammasome in airways from patients with COVID-19/B.1.1.7 variant. 

In addition, the tracheal NLRP3 mRNA expression in both COVID-19 variants correlated with C reactive protein (CRP) and lactate dehydrogenase (LDH) in serum as well as with the age of patients (Table 2).

Previous findings indicate that SARS-CoV-2 can activate the TLR2 innate immune pathway [16]. In this work, we observed an overexpression of TLR2 and its coupled MyD88 adaptor in tracheal tissue from COVID-19/B.1.1.7 patients (Figure 1; *p* < 0.0001 vs. COVID-19/B.1 patients), while only MyD88 was overexpressed in COVID-19/B.1 variant (Figure 1; *p* = 0.0077 vs. control patients).

The airway over-inflammation is accompanied by an increase in oxidative stress. In this work, we analyzed the balance between the nuclear factor erythroid 2-related factor 2 (Nrf2) antioxidant transcription factor and the nicotinamide adenine dinucleotide phosphate (NADPH) oxidase NOX4. Nrf2 was unaffected in COVID-19/B.1 variant but increased in COVID-19/B.1.1.7 variant (Figure 1; *p* < 0.0001 vs. COVID-19/B.1 patients), whilst NOX4 was upregulated in both COVID-19 variants. The ratio between NOX4 and Nrf2 expression was increased in both COVID-19 variants, which indicates that the protective effects of Nrf2 are outweighed by the oxidative effects of NOX4 (Figure 1). 

**Figure 2:**
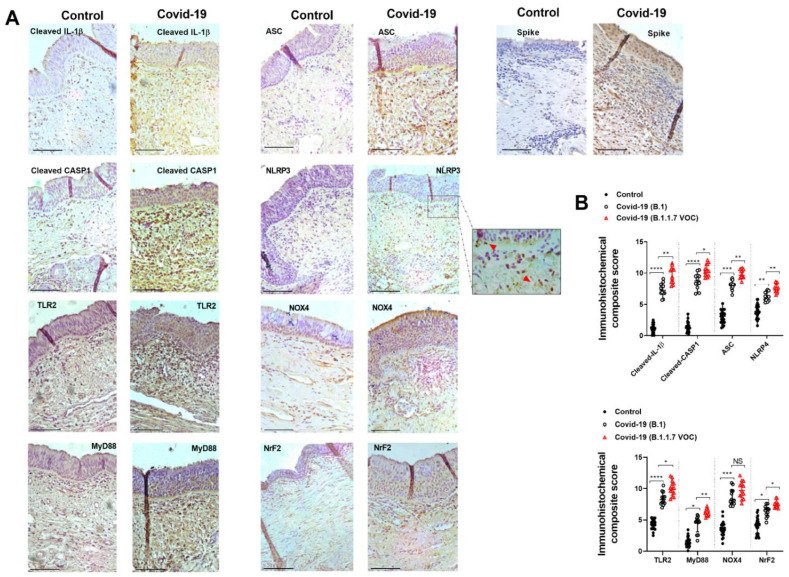
Inflammasome, innate immune and redox element immunohistochemical analysis in critically ill COVID-19 tracheal biopsies. Tracheal biopsies were obtained from invasive ventilated critically ill patients without COVID-19 and no respiratory disease/infections (control, *n* = 32), SARS-CoV-2 B.1 variant (*n* = 31), and B.1.1.7 VOC alpha variant (*n* = 20). (**A**) Tracheal biopsies were immune stained with indicated antibodies. Representative images are shown from different patients. (**B**) Immunohistochemical composite score quantification. Red arrows indicate NLRP3 punctate structure formation. Scale bar: 100 µm. Data are presented as scatter dot blots with median and interquartile range values. *p*-values are based on the Kruskal-Wallis test and Dunn’s post hoc test for multiple comparisons. * *p* < 0.05; ** *p* < 0.01; *** *p* < 0.001; **** *p* < 0.0001.

Immunohistochemical analysis showed similar results to that observed in gene expression evaluation (Figure 2A). The expression of inflammasome components (cleaved IL-1β, cleaved-CASP1, ASC, and NLRP3) was increased in COVID-19 variants and overexpressed in COVID-19/B.1.1.7 patients (Figure 2; *p* < 0.05 compared with COVID-19/B.1 variant) as indicates the immunohistochemical composite score (Figure 2B). The expression of inflammasome components was mainly located in infiltrated inflammatory cells in tracheal connective tissue and to a lesser extent, in the pseudostratified epithelium (Figure 2A). As observed in gene expression results, the expression of TLR2/MyD88 and NOX4/Nrf2 was also overexpressed in COVID-19 patients (Figure 2B). 

**Figure 3:**
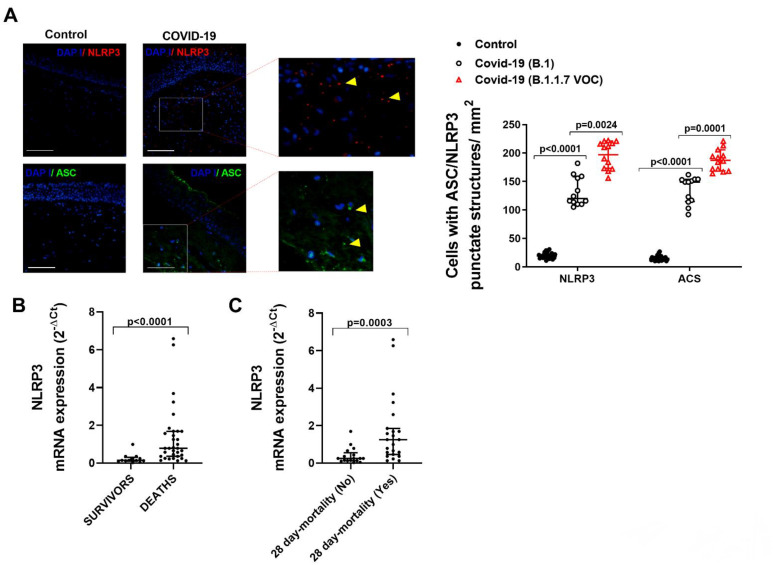
NLRP3/ASC inflammasome punctate structures formation and expression is increased in COVID-19 patients and associated with severity. Tracheal biopsies were obtained from invasive ventilated critically ill patients without COVID-19 and no respiratory disease/infections (control, *n* = 32), SARS-CoV-2 B.1 variant (*n* = 31), and B.1.1.7 VOC alpha variant (*n* = 20). (**A**) Immunofluorescence of NLRP3 and ASC in tracheal biopsies. Yellow arrows indicate inflammasome punctate structure formation. Scale bar: 100 µm. (**B**) mRNA expression of NLRP3 in survivors and deceased critically ill COVID-19 patients. (**C**) 28-day mortality: death within 28 days of hospital admission. Data are presented as scatter dot blots with median and interquartile range values. *p*-values are based on the Kruskal-Wallis test and Dunn’s post hoc test for multiple comparisons.

The activation of inflammasome requires the NLRP3 oligomerization, which recruits ASC protein to form inflammasome specks that can be visualized as punctate structures in immunofluorescence assays. Here, we observed an increased NLRP3/ASC punctate structures formation in COVID-19/B.1 patients (Figure 3A; *p* < 0.0001 vs. control patients) and overexpressed in COVID-19/B1.1.7 patients (Figure 3A; *p* < 0.0001 vs. COVID-19/B.1 patients). According to the increased inflammasome activation, we observed that NLRP3 mRNA expression was significantly overexpressed in the tracheal tissue of COVID-19 patients who died due to COVID-19 and in patients who died within 28 days of hospital admission (Figure 3B,C).

### 2.2. The Combination of SARS-CoV-2 Proteins S, E, and M Promotes the Release of Inflammatory Proteins in Human Macrophages and Bronchial Epithelial Cells

**Figure 4:**
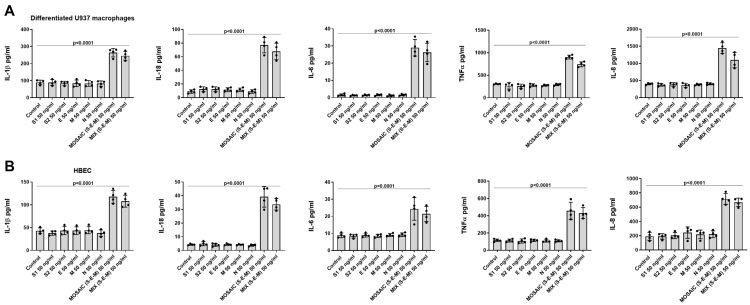
The combination of S, E, and M SARS-CoV-2 recombinant proteins induced inflammatory cytokine release in human differentiated U937 macrophages and bronchial epithelial cells. (**A**) Differentiated U937 macrophages and (**B**) human bronchial epithelial cells (HBEC) from two subjects were stimulated with SARS-CoV-2 recombinant proteins at 50 ng/mL, the combination of S1 + E + M recombinant proteins at 50 ng/mL each one or with the mosaic (S-E-M) recombinant protein for 24 h. Cell supernatants were collected, and cytokines were measured by ELISA. Data are presented as scatter dot blot of *n* = 4 independent experiments run in triplicate, with median and interquartile range values. *p*-values are based on the Kruskal-Wallis test and Dunn’s post-hoc test for multiple comparison.

Recombinant proteins of SARS-CoV-2, such as N, M, E, S1, and S2 glycoproteins, the combination of S, E, and M proteins and the S-E-M mosaic recombinant protein at a concentration of 50nM were used as a stimulus during 24 h in differentiated U934 macrophages and HBEC. Single recombinant proteins were not able to induce IL-1β, IL-18, IL-6, TNF-α, or IL-8 release in both cell types (Figure 4A,B). However, the combination of S, E, and M proteins or the S-E-M mosaic immunogenic recombinant protein at 50ng/mL were able to induce IL-1β, IL-18, IL-6, TNF-α and IL-8 release in both cell types (Figure 4A,B). Therefore, we used mosaic (S-E-M) recombinant protein for the rest of the experiments.

**Figure 5:**
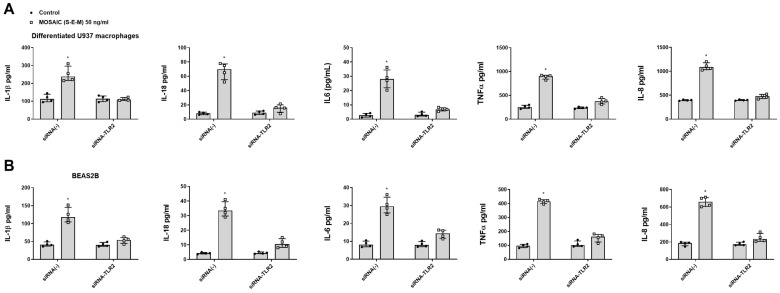
SARS-CoV-2 mosaic (S-E-M) recombinant protein mediates inflammatory responses through toll-like receptor 2 (TLR2). (**A**) Differentiated U937 macrophages and (**B**) bronchial epithelial cells BEAS2B were transiently transfected with control siRNA(-) or siRNA-TLR2 and stimulated with vehicle (control) or mosaic (S-E-M) for 24 h. Data are presented as scatter dot blot of *n* = 4 independent experiments run in triplicate, with median and interquartile range values. Statistical analysis was carried out using by Kruskal-Wallis test and Dunn’s post hoc test for multiple comparisons. * *p* < 0.05 vs. control.

Previous reports indicate that SARS-CoV-2 induction of pro-inflammatory proteins is mediated by TLR2 protein in human macrophages [28]. In this work, gene TLR2 silencing by transient siRNA-TLR2 suppressed the effects of mosaic (S-E-M) recombinant protein on inflammatory cytokine release in both human differentiated U937 macrophages (Figure 5A) and bronchial BEAS2B epithelial cells (Figure 5B) suggesting that innate immune activation of mosaic (S-E-M) recombinant protein is mediated by TLR2 signal. 

**Figure 6:**
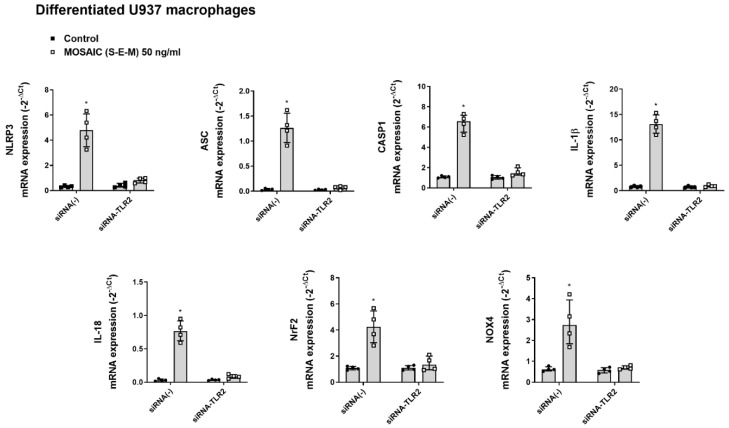
SARS-CoV-2 mosaic (S-E-M) recombinant protein mediates inflammasome and redox expression through toll-like receptor 2 (TLR2) in differentiated U937 macrophages. Differentiated U937 macrophages were transiently transfected with control siRNA(-) or siRNA-TLR2 and stimulated with vehicle (control) or mosaic (S-E-M) for 24 h. mRNA expression of target genes was measured by RT-qPCR. Data are presented as scatter dot blot of *n* = 4 independent experiments run in triplicate, with median and interquartile range values. Statistical analysis was carried out using by Kruskal-Wallis test and Dunn’s post hoc test for multiple comparisons. * *p* < 0.05 vs. control.

**Figure 7:**
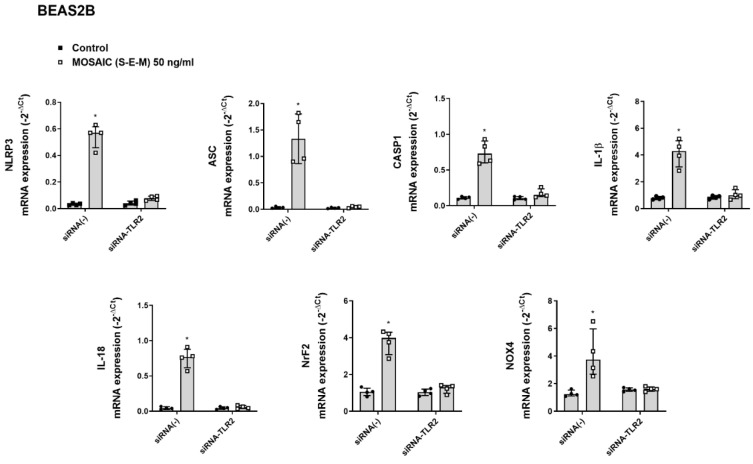
SARS-CoV-2 mosaic (S-E-M) recombinant protein mediates inflammasome and redox expression through toll-like receptor 2 (TLR2) in bronchial epithelial BEAS2B cells. BEAS2B cells were transiently transfected with control siRNA(-) or siRNA-TLR2 and stimulated with vehicle (control) or mosaic (S-E-M) for 24 h. mRNA expression of target genes was measured by RT-qPCR. Data are presented as scatter dot blot of *n* = 4 independent experiments run in triplicate, with median and interquartile range values. Statistical analysis was carried out using by Kruskal-Wallis test and Dunn’s post hoc test for multiple comparisons. * *p* < 0.05 vs. control.

In similar experiments, mosaic (S-E-M) recombinant protein increased the mRNA expression of the inflammasome components NLRP3, ASC, CASP1, IL-1β, and IL-18, as well as Nrf2 and NOX4 that were suppressed by transient siRNA-TLR2 transfection in differentiated U937 macrophages (Figure 6) and BEAS2B bronchial epithelial cells (Figure 7). 

### 2.3. N-acetylcysteine Inhibits SARS-CoV-2 Mosaic (S-E-M) Inflammation in a Concentration and Time-Dependent Fashion in Macrophages and Bronchial Epithelial Cells

**Figure 8:**
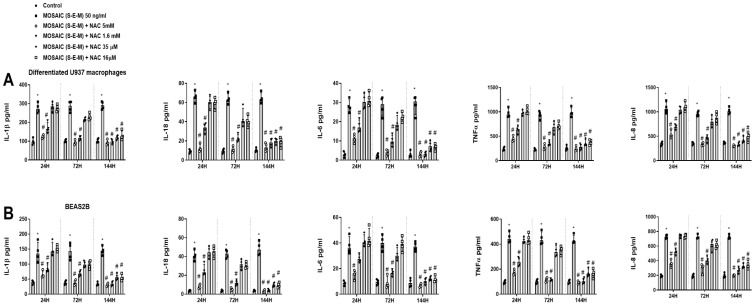
N-acetylcysteine inhibits concentration and time-dependent on the effects of SARS-CoV-2 mosaic (S-E-M) on inflammatory cytokines. (**A**) Differentiated U937 macrophages and (**B**) bronchial epithelial BEAS2B were incubated with N-acetylcystine (NAC) at 16 µM, 35 µM, 1.6 mM, and 5 mM for 24 h, 72 h, and 144 h, followed by the stimulation with mosaic (S-E-M) 50 ng/mL for 24 h. Cell supernatants were collected to measure different cytokines by ELISA. Data are presented as scatter dot blot of *n* = 4 independent experiments run in triplicate, with median and interquartile range values. Statistical analysis was carried out using by Kruskal-Wallis test and Dunn’s post hoc test for multiple comparisons. * *p* < 0.05 vs. control. ^#^ *p* < 0.05 vs. mosaic (S-E-M) stimulus.

N-acetylcysteine (NAC) concentrations of 16 µM, 35 µM, 1.6 mM, and 5 mM were added for 24 h, 72 h, and 144 h, followed by the stimulation with SARS-CoV-2 mosaic (S-E-M) recombinant protein for another 24 h. After 24 h and 72 h of NAC pre-incubation, only the 1.6 mM and 5 mM NAC concentrations showed a robust inhibitory effect on the mosaic (S-E-M)-induced IL-1β, IL-18, IL-6, TNF-α, and IL-8 secretion in differentiated U937 macrophages (Figure 8A) and BEAS2B cells (Figure 8B). In addition, NAC at 16 µM and 35 µM concentrations showed inhibitory effects on the mosaic (S-E-M)-induced pro-inflammatory cytokines after 144 h of pre-incubation, suggesting that low concentrations of NAC in a prolonged period of time have anti-inflammatory properties (Figure 8A,B). 

**Figure 9:**
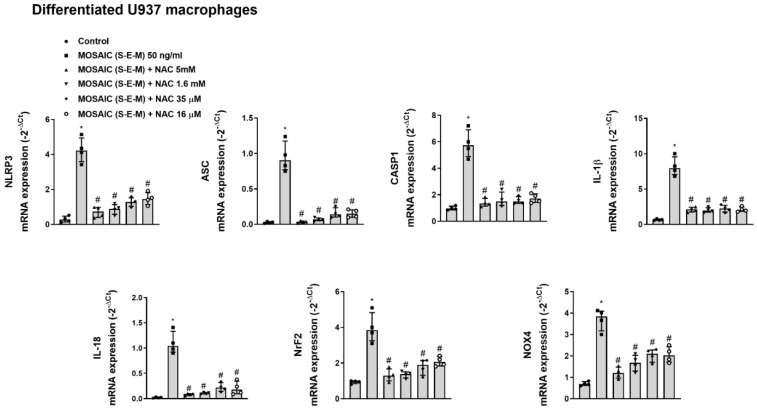
N-acetylcysteine inhibits the effects of SARS-CoV-2 mosaic (S-E-M) on inflammasome and redox mRNA expression markers in differentiated U937 macrophages. Differentiated U937 macrophages were incubated with N-acetylcystine (NAC) at 16 µM, 35 µM, 1.6 mM, and 5 mM for 144 h, followed by the stimulation with mosaic (S-E-M) 50 ng/mL for 24 h. mRNA expression of target genes was measured by RT-qPCR. Data are presented as scatter dot blot of *n* = 4 independent experiments run in triplicate, with median and interquartile range values. Statistical analysis was carried out using by Kruskal-Wallis test and Dunn’s post hoc test for multiple comparisons. * *p* < 0.05 vs. control. ^#^ *p* < 0.05 vs. mosaic (S-E-M) stimulus.

**Figure 10:**
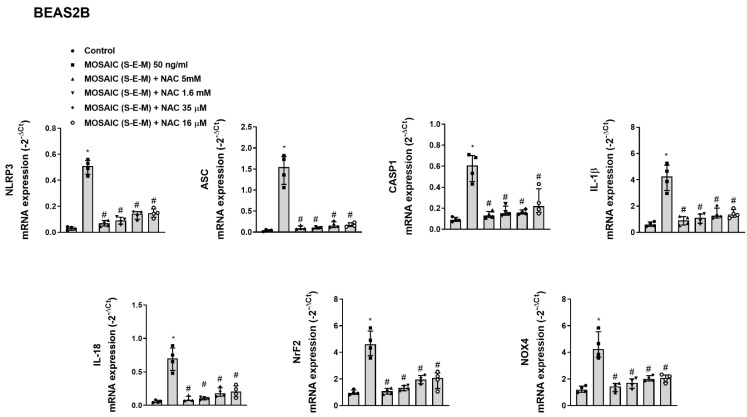
N-acetylcysteine inhibits the effects of SARS-CoV-2 mosaic (S-E-M) on inflammasome and redox mRNA expression markers in bronchial epithelial BEAS2B cells. BEAS2B cells were incubated with N-acetylcystine (NAC) at 16 µM, 35 µM, 1.6 mM, and 5 mM for 144 h, followed by the stimulation with mosaic (S-E-M) 50 ng/mL for 24 h. mRNA expression of target genes was measured by RT-qPCR. Data are presented as scatter dot blot of *n* = 4 independent experiments run in triplicate, with median and interquartile range values. Statistical analysis was carried out using the Kruskal-Wallis test and Dunn’s post hoc test for multiple comparisons. * *p* < 0.05 vs. control. ^#^ *p* < 0.05 vs. mosaic (S-E-M) stimulus.

Similarly, the incubation of NAC for 144 h at all concentrations assayed 16 µM, 35 µM, 1.6 mM, and 5 mM inhibited the effect of mosaic (S-E-M) on NLRP3, ASC, CASP1, IL-1β, and IL-18 inflammasome mRNA expression as well as the mRNA expression of Nrf2 and NOX4 in differentiated U937 macrophages (Figure 9) and BEAS2B epithelial cells (Figure 10). 

### 2.4. N-acetylcysteine Inhibits the SARS-CoV-2 Mosaic (S-E-M)-Induced Inflammasome Activation and Intracellular Nf-κB and ERK1/2 Inflammatory Pathways

**Figure 11:**
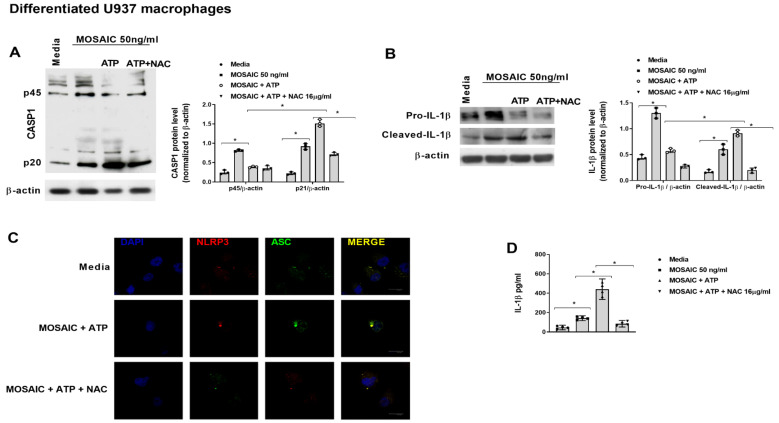
N-acetylcysteine inhibits inflammasome activation in human-differentiated U937 macrophages. (**A**–**D**) Differentiated U937 macrophages were incubated with N-acetylcysteine (NAC) at 16 µM for 144 h, followed by the stimulation with recombinant SARS-CoV-2 mosaic (S-E-M) protein in the presence of NAC for 24 h. At the end of stimulation, ATP 5 mM was added for 1 h to stimulate NLRP3 assembly and activation. (**A**) Protein expression of different forms of CASP1 and (**B**) IL-1β were analyzed by western blot and quantified by densitometry. Representative western blots are shown from *n* = 3 independent experiments. (**C**) Immunofluorescence analysis of inflammasome NLRP3/ASC punctate structures formation. (**D**) Cell supernatant levels of IL-1β were measured by ELISA. Data are presented as scatter dot blot of *n* = 3–4 independent experiments run in triplicate, with median and interquartile range values. Statistical analysis was carried out by Kruskal-Wallis test and Dunn’s post hoc test for multiple comparisons. * *p* < 0.05.

To study the effects of NAC on the mosaic (S-E-M)-induced inflammasome activation, differentiated U937 macrophages were incubated with NAC 16 µM for 144 h followed by the stimulation with recombinant mosaic (S-E-M) SARS-CoV-2 protein in the presence of NAC for 24 h. At the end of stimulation, ATP 5 mM was added for 1h to stimulate NLRP3 assembly and activation. In these conditions, NAC was able to reduce the formation of the short CASP1 p20 active form (Figure 11A), as well as the consequent cleaved-IL-1β active form (Figure 11B), which was translated in a reduction in an NLRP3/ASC punctate structures formation (Figure 11C) and reduced secretion of IL-1β (Figure 11D). 

**Figure 12:**
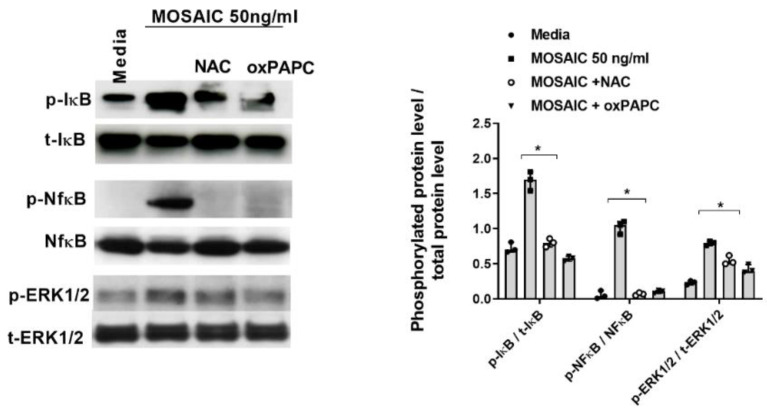
N-acetylcysteine inhibits the effects of SARS-CoV-2 mosaic (S-E-M) protein on Nf-κB and ERK1/2 activation. Differentiated U937 macrophages were incubated with N-acetylcysteine (NAC) at 16 µM for 144 h or with the TLR2 inhibitor oxPAPC 30 µg/mL for 1 h, followed by the stimulation with recombinant SARS-CoV-2 mosaic (S-E-M) protein for 24 h. Protein expression was measured by western blot. Representative blots are shown from *n* = 3 independent experiments. Data are presented as scatter dot blots with median and interquartile range values. Statistical analysis was carried out by Kruskal-Wallis test and Dunn’s post hoc test for multiple comparisons. * *p* < 0.05.

In other experiments, differentiated U937 cells were pre-incubated with NAC 16 µM for 144 h or with the TLR2 inhibitor oxPAPC at 30µg/mL for 1 h, followed by the stimulation with mosaic (S-E-M) SARS-CoV-2 protein during 30 min and 60 min. Both NAC and oxPAPC reduced the mosaic (S-E-M)-induced IκB and NfκB phosphorylations at 60 min of stimulation and ERK1/2 phosphorylation after 30 min of stimulation (Figure 12). 

### 2.5. Effects of Mosaic (S-E-M) SARS-CoV-2 Protein on Oxidative Stress, Thiols, and Glutathione

**Figure 13:**
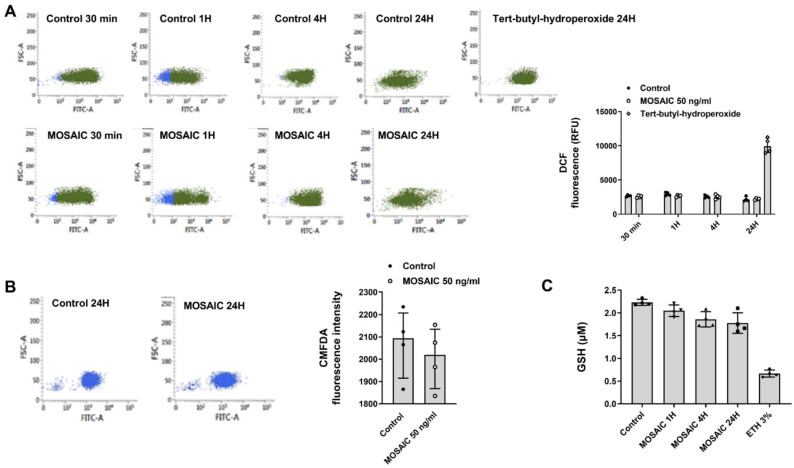
SARS-CoV-2 mosaic (S-E-M) recombinant protein has no effect on oxidative stress, thiols, and GSH in differentiated U937 macrophages. Differentiated U937 macrophages were stimulated with SARS-CoV-2 mosaic (S-E-M) 50 ng/mL for the indicated time points. (**A**) Reactive oxygen species were measured by flow cytometry and quantified by DCF relative fluorescence units. Representative plots are shown. (**B**) Thiol levels were measured by flow cytometry, and (**C**) GSH was measured by glutathione Assay kit as described in the methods. Data are presented as scatter dot blot of *n* = 4 independent experiments with median and interquartile range values. Statistical analysis was carried out by Kruskal-Wallis test and Dunn’s post hoc test for multiple comparisons.

Previous evidence showed that NAC could enhance the endogenous production of H2S and replenish reduced intracellular glutathione (GSH) pools in addition to direct scavenger effects on reactive oxygen species (ROS) [29]. However, there is no evidence of the direct effects of SARS-CoV-2 proteins on H2S, GSH, and ROS. In this work, we used the mosaic (S-E-M) SARS-CoV-2 recombinant protein at 50 ng/mL concentration that showed pro-inflammatory properties to analyze the effects on oxidative stress in differentiated U937 macrophages. In this regard, mosaic (S-E-M) did not increase the levels of intracellular ROS measured as DCF fluorescence at different time points (1–24 h; Figure 13A). In addition, mosaic (S-E-M) did not modify intracellular thiols (Figure 13B) and GSH (Figure 13C), although it showed a trend toward decrease. These results suggest that NAC has direct inhibitory effects on SARS-CoV-2 by targeting inflammatory pathways rather than oxidative stress.

## 3. Discussion

The present work shows novel evidence of the effects of SARS-CoV-2 on airway inflammasome activation in critically ill COVID-19 patients, as well as the correlation between the increased inflammasome activation and COVID-19 severity and mortality. In vitro, we showed that the combination of recombinant S, E, and M SARS-CoV-2 proteins were able to activate the inflammasome in macrophages and bronchial epithelial cells, increasing the release of pro-inflammatory mediators through a mechanism that includes TLR2/MyD88/NfκB/ERK1/2 activation. In addition, we showed that large exposures of low in vitro concentrations of NAC of 16 µM and 35 µM, corresponding with plasma levels achieved by oral 600 mg and 1200 mg administrations [30,31], were able to inhibit inflammasome activation and inflammation, which may be of potential value as adding scientific knowledge to support the chronic use of NAC in COVID-19 patients.

In this work, we selected live critically ill patients with COVID-19 B.1 and B.1.1.7 VOC variants to study the activation of inflammasome in airways at the moment of tracheostomy, and its association with airway inflammation, systemic inflammation, hospital mortality, and 28-day mortality. According to the epidemiologic data, the SARS-CoV-2 B.1.1.7 VOC (alpha variant) provoked an elevated number of COVID-19 cases with higher mortality than that observed with the first B.1 variant in Valencia, Spain [32]. The clinical data of patients included in this work showed an increased age, 28-day mortality, hypertension, and serum inflammatory parameters such as CRP and LDH in the B.1.1.7 VOC patients compared with B.1 variant. All of these clinical parameters haven been previously correlated with high mortality [33] in COVID-19 patients. In this line, the expression of inflammasome components, as well as the activation of inflammasome measured as NLRP3/ASC punctate structures in tracheal tissue, was more elevated in B.1.1.7 VOC variant than in B.1 variant and correlated with the CRP and LDH serum values which indicate a more severe inflammation which could be driven by inflammasome activation. Interestingly, aging has been connected with a susceptible elevation of inflammasome activation, namely inflammation [34], consisting of sustained low levels of chronic inflammation due to old age that could prime and predisposes COVID-19-older patients to an overactivation of the inflammasome, explaining, almost in part, the higher mortality in aged patients. This hypothesis was supported in this work by the fact that NLRP3 expression in airway tissue was positively correlated with the age of patients.

Previous reports suggest that SARS-CoV-2 infection senses NRLP3 [11,13] and that increased levels of IL-1β and IL-18 in plasma correlate with disease severity and mortality in patients with COVID-19 [35]. In this regard, SARS-CoV-2 in vitro infection can induce NLRP3 activation in human peripheral blood monocytes, and NLRP3 is activated in blood monocytes from COVID-19 patients. In addition, NLRP3 activation has been demonstrated in postmortem lung tissue of COVID-19 patients, a condition that could modify the NLRP3 expression and activation [11]. However, to our knowledge, there is no data on NLRP3 expression and activation in airway tissue of live COVID-19 patients and no data regarding the association of the airway NLRP3 inflammasome and COVID-19 severity and mortality. In this work, we presented the first evidence on NLRP3 status in airway tissue in different SARS-CoV-2 variants as well as its positive association with the disease severity and mortality, which represents an increase in the knowledge of pathomechanism linked to COVID-19 disease.

Previous reports showed that PAMPs associated with SARS-CoV-2, such as single-stranded RNA [36], ORF3a, and the N protein, could activate NLRP3 inflammasome [37,38] in vitro. In addition, it has been shown that the SARS-CoV-2 E protein promotes TLR2 signaling but not TLR4, which upregulates the expression of NLRP3 and IL-1β in macrophages [28]. However, other authors showed that recombinant SARS-CoV-2 S protein increased NLRP3 protein expression and induction of IL-1β in macrophages from patients with COVID-19 through the up-stream activation of TLR2 [32,39]. To increase the complexity, in silico analysis has shown that TLR1, TLR4, and TLR6 bind S protein [40] and that S protein activates TLR4 in human macrophages [41,42]. In terms of mechanism of action, one explanation could be that SARS-CoV-2 causes an imbalance in intracellular potassium efflux to activate NLRP3 inflammasome and IL-1β and IL-18 release [13]. Altogether, current data is controversial, and the role of specific SARS-CoV-2 proteins in triggering inflammasome remains to be characterized. In this work, we use low concentrations of 50ng/mL of different SARS-CoV-2 proteins that did not produce IL-1β and IL-18 release. However, the combination of S, E, and M proteins showed synergic effects activating inflammasome IL-1β and IL-18 release as well as the pro-inflammatory cytokines IL-6, TNF-α, and IL-8. This protein combination appears to have the potential to induce an immune innate inflammatory response since the SARS-CoV-2 mosaic (S-E-M) reproduced a similar inflammatory response to the whole S, E, and M protein combination. In this regard, SARS-CoV-2 mosaic (S-E-M) recombinant protein, which contains SARS-CoV-2 S, M, and E immunodominant regions, fused to His tag at C-terminal, was selected in this work as a stimulus in vitro model approximation, for the mechanistic analysis of SARS-CoV-2 innate immune inflammation. The latter option represents a limitation of this study since recombinant proteins did not allow to reproduce of the whole structure of the SARS-CoV-2 virus, including its spatial 4D conformation, which may limit the interpretation of the results of this manuscript. Similar limitations have been described for established in vitro viral/bacterial models such as poly I:C intracellular dsRNA [43], a viral TLR3 agonist, or synthetic triacylated lipopeptide Pam3CSK4 (Pam3CysSerLys4) as a TLR2/TLR1 ligand [44]. 

In this work, we observed that the SARS-CoV-2 mosaic (S-E-M) increased the inflammasome NLRP3/ASC/CASP1 axis and IL-1β and IL-18 release through the activation of TLR2 signaling. In terms of innate immunity, we showed that TLR2 and its adaptor MyD88 were overexpressed in tracheal biopsies of COVID-19 patients, which could contribute to the exaggerated inflammasome activation in COVID-19. 

The hyper-inflammatory syndrome of severe COVID-19 patients is accompanied by elevated oxidative stress [45]. In fact, serum levels of antioxidant thiols and GSH, among others, are decreased and inversely correlated with COVID-19 severity, while reactive nitric and oxygen species are overexpressed and correlated with COVID-19 severity [45]. In this work, we evaluated the expression and the balance between the anti-oxidant transcription factor Nrf2 and the oxidant NOX4 cell membrane protein since the increase in NOX4/Nrf2 ratio has been associated with lung fibrosis [46], important sequelae of COVID-19 [47]. We observed an increase in Nrf2 and NOX4 expression in tracheal epithelial cells and infiltrated inflammatory cells, but an increased NOX4/Nrf2 redox imbalance, suggesting that the overexpression of the reactive oxygen species-generating enzyme NOX4 overcame the capacity to induce the Nrf2 as previously outlined in patients with lung fibrosis [46]. In physiological conditions, the increase in oxidative stress elevates the activation of Nrf2 as a compensatory mechanism. In contrast, COVID-19 positive patients show decreased Nrf2 serum levels and total antioxidant status, and elevated NOX4 serum levels suggesting that SARS-CoV-2 could inactivate the antioxidant Nrf2 [48,49]. However, these results have been described in serum samples, and no data exist in airway tissue and inflammatory cells. In this work we detected elevated levels of Nrf2 in COVID-19 patients compared with non-COVID-19 patients and observed that SARS-CoV-2 mosaic (S-E-M) protein increases the expression of both Nrf2 and NOX4 in macrophages and bronchial epithelial cells in vitro, through the activation of TLR2 receptor. However, we did not detect a direct increase in intracellular reactive oxygen species (ROS) induced by mosaic (S-E-M). In addition, although mosaic (S-E-M) protein slightly decreased thiols and GSH levels, this decrease was not statistically significant. An explanation of these apparently contradictory results could be discussed by the fact that SARS-CoV-2 produces an exaggerated inflammatory response, and these inflammatory proteins elevate ROS levels that are able to increase inflammation, thus producing a positive feedback loop. In this regard, ROS can activate Nf-κB and NLRP3, thus elevating the even more, the inflammation [45]. Although we have observed induction of NOX4 by mosaic (S-E-M) after 24 h of stimulation, the experimental conditions and ROS readouts (up to 24 h), together with the elevation of Nrf2, can explain the undetectable in vitro ROS production.

In this context, the increased inflammation and the redox imbalance in severe COVID-19 patients have aroused the interest of scientists for different anti-inflammatory and potential antioxidant treatments. In this regard, NAC has been postulated as a potential treatment for COVID-19 because of the antioxidant, anti-inflammatory, and anti-viral properties. In COVID-19 patients, high doses of NAC (600–1200 mg/day) may prevent the severe symptoms of COVID-19 in observational and case-report studies, although negative results have also been reported in interventional studies, which have encouraged scientists to conduct a broad number of NAC clinical trials in COVID-19 patients which are currently ongoing [45]. Mechanistically, NAC is a direct ROS scavenger, a source of sulfhydryl (SH) groups that elevates thiol groups and increases levels of GSH. It also modulates several signaling pathways such as p38, ERK1/2, SAPK/JNK, c-Jun, and c-Fos [50] and exerts some anti-inflammatory effects via inhibition of Nf-κB [51]. In addition, it has been shown that NAC inhibits NLRP3 activation [52]. I t is known that ROS activates Nf-κB that induces NLRP3 expression. In addition, ROS can directly promote NLRP3 assembly and activation [45]. In this work, we showed for the first time the effects of NAC on the inhibition of SARS-CoV-2 mosaic (S-E-M) protein-induced NLRP3/ASC/CASP1 and IL-1β/IL-18 inflammasome activation as well as the inhibition of pro-inflammatory cytokines IL-6, IL-8 and TNFα in human macrophages and bronchial epithelial cells. The effects of NAC on SARS-CoV-2 mosaic (S-E-M)-induced NLRP3 activation were mediated by Nf-κB inhibition, but also by direct effects since NAC inhibited the ATP-induced NLRP3/ASC/CASP1/IL-1β activation. Previous reports have found that physiological concentrations of thiols inhibit the activity of the NLRP3 inflammasome and reduce the production of pro-inflammatory cytokines in vitro and in vivo [53,54]. Therefore, the inhibitory effects of NAC on SARS-CoV-2 mosaic (S-E-M)-induced NLRP3 activation could be mediated by the increase in thiol levels. In addition, SARS-CoV-2 virus has demonstrated to activate canonical inflammasome, including gasdermin D N-terminal which form a cell membrane pore to release IL-1β and IL-18 outside the cell, as well as cell pyroptosis [55,56]. In addition, NAC inhibited inflammasome and gasdermin D activation induced by Bisphenol A [57] or arsenic trioxide [58] in osteocytes and hepatocytes respectively. In this work, NAC inhibited the SARS-CoV-2 mosaic (S-E-M)-induced CASP1 activation that is necessary to cleave and activate gasdermin D, therefore it is reasonable to think that N-acetylcyste inhibits SARS-CoV-2 mosaic (S-E-M) -induced Gasdermin D activation. Future works must be explored these hypotheses in detail.

In this work, we did not observe an increase in ROS after mosaic (S-E-M) stimulation, so the anti-inflammatory effects of NAC observed in this work can be independent of its antioxidant properties. In fact, previous works have shown the inhibitory effects of NAC on Nf-κB activation independently of its antioxidant effects [59]. In addition to the mechanisms studied in this work, a computational study showed that thiol-based chemicals could shift the spike glycoprotein redox scaffold of SARS-CoV-2, inhibiting the SARS-CoV-2 infection [60].

Other important novel results of the present work are that we probe that chronic low in vitro concentrations of NAC can inhibit inflammasome. Regarding the in vitro results, there are some controversies because the efficient doses used in the scientific literature are usually higher (mM concentrations) than the achieved in vivo plasma concentrations (µM concentrations) [29,61]. These differences are explained by the possibility that in vivo, NAC treatment for a prolonged time maintains redox-dependent cell signaling and transcription [62]. Therefore, we suggested that, similar to what happens in vivo, low and high NAC doses can exert anti-inflammatory activities, but their effectiveness is influenced by the duration of treatment. In this regard, we showed that chronic in vitro exposures of NAC 16 µM and 35 µM, corresponding to plasma levels following 600 mg and 1200 mg of oral NAC [30,31], showed the same anti-inflammatory effects that the common in vitro short-period NAC concentrations (1.6 mM and 5 mM) used in the literature, which may be more representative in terms of translational information requiring large periods of NAC administration in COVID-19 patients. In summary, we showed novel evidence on the activation of inflammasome in critically ill COVID-19 patients, as well as the correlation with NLRP3 activation and severity. The combination of SARS-CoV-2 S-E-M proteins activates TLR2 signaling increasing Nf-κB/ERK1/2 and inflammasome activation, which is attenuated by NAC, suggesting a plausible explanation of the beneficial effects of NAC observed in COVID-19 patients.

## 4. Materials and Methods

### 4.1. Patients

Critically ill patients diagnosed with COVID-19 were recruited in the Valencia General University Hospital Consortium, Spain, in the first COVID-19 wave (24 February to 20 June 2020) characterized by the Sars-CoV-2 B.1 variant, and in the third wave period (6 December 2020 to 13 March 2021) characterized by the B.1.1.7 VOC (Alpha variant). The following inclusion criteria were applied: (A) Major Criteria: (1) need for invasive mechanical ventilation, (2) shock (need for vasoactive drugs), and (B) Minor Criteria: (1) respiratory rate >30 rpm, (2) PaFiO2 <150, (3) multilobular infiltrates, (4) confusion, (5) disorientation, (6) uraemia >20 mg/dl, (7) leukopenia <4000/mm^3^, (8) thrombopenia <100,000/mm^3^, (9) temperature <36.8 °C, (10) arterial hypotension requiring aggressive fluid therapy. In patients with COVID-19 admitted to the critical care unit, intubated patients were undergoing tracheostomy around the 10th day of intubation, with the intention of avoiding laryngeal injuries due to decubitus caused by the tube. A tracheal fragment was resected which included 1–2 tracheal rings depending on the patient’s anatomy. The tracheal fragment, which measures approximately 15 × 5–7 mm was processed to extract total RNA in protected conditions or preserved in formaldehyde for later processing. Critically ill patients with negative PCR for SARS-CoV-2 and without respiratory tract infection were used as control patients. Clinical characteristics of critically ill COVID-19 patients are described in Table 1. The protocol was approved by the local research and independent ethics committee of the University General Consortium Hospital of Valencia (CEIC60/2020). Informed written consent was obtained from each participant.

### 4.2. Real-Time qRT-PCR and Gene Silencing

Total RNA was isolated using TriPure^®^ Isolation Reagent (Roche, Indianapolis, IN, USA). The integrity of the extracted RNA was confirmed with Bioanalyzer (Agilent, Palo Alto, Santa Clara, CA, USA). Reverse transcription was performed in 300 ng of total RNA with a TaqMan reverse transcription reagents kit (Applied Biosystems, Perkin-Elmer Corporation, Foster City, CA, USA). cDNA was amplified with specific primers and probes predesigned by Applied Biosystems for humans: IL-1β (Hs01555410_m1), IL-18 (Hs01038788_m1), NLRP3 (Hs00918082_m1), ASC (Hs00203118_m1), CASP1 (Hs00354836_m1), TLR2 (Hs00610101_m1), MyD88 (Hs00182082_m1), Nrf2 (Hs00975960_m1), NOX4 (Hs01379108_m1), in a 7900HT Fast Real-Time PCR System (Applied Biosystems) using Universal Master Mix (Applied Biosystems). Expression of the target gene was expressed as the fold increase or decrease relative to the expression of β-actin as an endogenous control (Applied Biosystems; Hs01060665). The mean value of the replicates for each sample was calculated and expressed as the cycle threshold (Ct). The level of gene expression was then calculated as the difference (ΔCt) between the Ct value of the target gene and the Ct value of β-actin. The fold changes in the target gene mRNA levels were designated 2^−ΔCt^.

Small interfering RNA (siRNA), including the scrambled siRNA control (identification no. 4390843), was purchased from Ambion (Huntingdon, Cambridge, UK). TLR2 (identification no. s107052) gene-targeted siRNA was designed by Ambion. Monocytes and human bronchial epithelial cells (HBEC) were transfected with siRNA (50 nM) in serum and antibiotic-free medium. After 6 h, the medium was aspirated and replaced with medium containing serum for a further 42 h before cell stimulation. The transfection reagent used was lipofectamine-2000 (Invitrogen, Paisley, UK) at a final concentration of 2 µg/mL. TLR2 knockdown was evaluated in human monocytes and HBEC by RT-PCR using TLR2 primers and the probe described above. TLR2 expression in TLR2 knockdown experiments was always lesser than the 10% of TLR2 expression in siRNA(-) control cells.

### 4.3. Immunohistochemical and Immunofluorescence Studies

Tracheal histology was conducted as previously reported [63]. For immunohistochemical analysis of the human trachea, tissue was fixed, embedded in paraffin, and cut into sections (4–6 µm). Tracheal sections were immuno-stained with rabbit cleaved-IL-1β (Asp116) (D3A3Z) (cell signaling, cat. no. 83186S), rabbit cleaved caspase-1 (Asp297) (D57A2) (cell signaling, cat. no. 4199), rabbit NLRP3 (D4D8T) (cell signaling, cat. no. 15101), rabbit ASC/TMS1 (E1E3I) (cell signaling, cat. no. 13833), mouse TLR2 (Novus Biologicals, cat. no NBP1-51792B), rabbit MyD88 (D80F5) (cell signaling, cat. no. 4283), rabbit NOX4 (Novus Biologicals, cat. no NB110-58849), rabbit Nrf2 (Novus Biologicals, cat. no NBP1-32822) and rabbit SARS-CoV-2 Spike (Abcam, cat. no. ab272504, Cambridge, UK) for 24 h at 4 °C. A secondary Master polymer plus detection system (peroxidase) (incl. dab chromogen, Master diagnostica, cat. no. MAD-000237QK) was used for immunohistochemistry. The non-immune rabbit IgG isotype control (Sigma Aldrich cat. no DUO87004, St. Louis, MO, USA) was used as negative control and resulting in negative staining for all samples. 

Stained slices were scored by a pathologist in a blinded fashion under a Nikon Eclipse TE200 light microscope (Tokio, Japan), and representative photographs were taken (10 slices per patient). Staining intensity was analyzed in tracheal epithelium and mucosa. Staining intensity for different antibodies was scored on a scale of 0 to 3 (0, negative; 1, weak; 2, moderate; 3, strong immunoreactivity). The percentage of cells positive for different antibodies within the human trachea was scored on a scale of 1 to 4 as follows: 1, 0–25% cells positive; 2, 26–50% positive; 3, 51–75% positive; and 4, 76–100% positive. The score of the staining intensity and percentage of immunoreactive cells were then multiplied to obtain a composite score ranging from 0 to 12. 

Immunofluorescence analysis was performed to detect NLRP3 and ASC punctate structures expression and distribution in the human trachea. Trachea tissue was fixed in paraformaldehyde (4%) for 48 h, and tissue was embedded in Tissue-Tek^®^ OCT™ cryosectioning compound (Sakura Finetek Europe BV, Leiden). Blocks were cut into 10µm thick sections, permeabilized in Triton X 100 (0.1% in PBS) for 5 min, blocked in 10% goat serum in PBS, and immunostained with rabbit NLRP3 (D4D8T) (cell signaling, cat. no. 15101) or rabbit ASC/TMS1 (E1E3I) (cell signaling, cat. no. 13833) for 24 h at 4 °C followed by a secondary FITC or rhodamine-conjugated anti- rabbit IgG antibody and finally DAPI (2 µg/mL) to mark nuclei (Molecular Probes, Leiden, The Netherlands). Quantification of the number of NLRP3 and ASC punctate structures per mm^2^ was performed in 10 slices per patient using a confocal spectral Leica TCS SP2 microscope with ×600 magnification and 3× zoom. Red (HeNe 543 nm), green (HeNe 488 nm), and blue (Ar 351 nm, 364 nm) lasers were used.

### 4.4. Isolation and Culture of Human Bronchial Epithelial Cells and Mononuclear Cells

Isolation of human bronchial epithelial cells was assessed as previously outlined [64]. Small pieces of human bronchi (0.5–1 mm internal diameter) were excised from microscopically normal lung areas, carefully dissected free from lung parenchyma, and plated on collagen-coated culture dishes (10 µg cm^−2^ rat type I collagen (Sigma) in bronchial epithelial growth medium (BEGM, comprising of bronchial epithelial basal medium (BEBM) supplemented with single quotes (Lonza bioscience, Madrid, Spain)). Small bronchi were oriented with the epithelial layer to be in contact with the culture plate. After a period of ~1 week-12 days, bronchial epithelial cells were observed around the bronchi. The identity of the monolayer as bronchial epithelial cells was affirmed as previously, outlined [65]. Cell viability was assessed by vital trypan blue exclusion analysis using the countness^®^ automated cell counter (life technologies, Madrid, Spain). Cell viability was >98% in all cell cultures tested in this work. The bronchial epithelial BEAS2B cell line was obtained from American Type Culture Collection and cultured in BEGM media with supplements (Lonza, Madrid, Spain) on collagen-coated culture dishes (10 µg cm^−2^; rat type I collagen) at 37 °C with 5% CO_2_ in humidified air. The culture medium was replaced every 48 h.

The human monocytic cell line U937 (American Type Culture Collection, Rockville, MD, USA) was cultured in RPMI 1640 medium (Sigma-Aldrich Co., St Louis, MO, USA) supplemented with 10% fetal bovine serum (FCS), 100 U/mL penicillin and 100 mg/mL streptomycin at 37 °C with 5% CO_2_ in a humidified incubator. U937 cells were incubated at 37 °C with 5 nM of 12-O-tetradecanoylphorbol-13-acetate (TPA; Sigma-Aldrich Co.). The U937 monocyte cell line was differentiated after a 72 h incubation with TPA to macrophage-like cells as previously we outlined [66]. Under these conditions, the macrophage-like phenotype was proven based on cell surface markers such as the down-regulation of CD14 as well as the up-regulation of NaF-insensitive esterase and acid phosphatize [67]. Cells were centrifuged and resuspended in fresh media in 24-well plates at a concentration of 500 × 103 cells per well for 24 h before experimental use.

### 4.5. In Vitro Stimulations and Conditions

For in vitro studies, HBEC and differentiated 937 macrophages were stimulated with different recombinant proteins of SARS-CoV-2, (1) recombinant SARS-CoV-2 nucleocapsid (N) protein (Abcam, cat. no. ab273530), (2) recombinant SARS-CoV-2 membrane (M) protein (mybiosource, cat. no. MBS8574735), (3) recombinant SARS-CoV-2 envelope (E) protein (Abclonal, cat. no. RP01263LQ-100), (4) recombinant SARS-CoV-2 Spike Glycoprotein S1 (Abcam, ab272105), (5) recombinant SARS-CoV-2 Spike Glycoprotein S2 (Novus Biologicals, NBP2-90973) and (6) recombinant SARS-CoV-2 mosaic S-E-M recombinant protein (BioVendor R&D, cat. no. RP9720140250; which is an Echerichia Coli derived recombinant protein containing the SARS-CoV-2 spike (S), membrane (M), and envelope (E) immunodominant regions, fused to His tag at C-terminal) for the indicated times. 

Cells were seeded and incubated with oxidized PAPC at 30µg/mL (oxPAPC, TLR2 antagonist [28], InVivoGen, cat. no. tlrl-oxp1) or with increasing N-acetyl-L -cysteine (NAC; Sigma: catalog no. A-7250) concentrations of 16 µM, 35 µM, 1.6 mM and 5mM for 24 h, 72 h and 144 h followed by the stimulation with SARS-CoV-2 recombinant proteins. After 24 h of cell stimulation, the culture medium was collected, and IL-1β, IL-18, IL-6, TNFα, and IL-8 were measured using commercially available Quantikine^®^ ELISA kits (R&D Systems, Madrid, Spain) according to the manufacturer’s protocol. 

In other experiments, differentiated U937 macrophages were incubated with NAC 16 µM for 144 h (replacing culture medium and NAC each 24 h), followed by the stimulation with recombinant SARS-CoV-2 MOSAIC protein in the presence of NAC for 24 h. At the end of stimulation, ATP 5 mM (Sigma Aldrich, cat. no. A6419) was added for 1h to stimulate NLRP3 assembly and activation.

### 4.6. Western Blot

Western blotting analysis was used to detect changes in human differentiated U937 macrophage protein expression. Cells were homogenized from a confluent 25-cm^2^ flask and lysed on ice with a lysis buffer comprising a complete inhibitor cocktail plus 1 mM ethylenediaminetetra acectic acid (Roche Diagnostics Ltd., West Sussex, UK) with 20 mM Tris base, 0.9% NaCl, 0.1% Triton X-100, 1 mM dithiothreitol, and 1 mg/mL pepstatin A. The Bio-Rad assay (Bio-Rad Laboratories Ltd., Herts, UK) was used according to the manufacturer’s instructions to quantify the level of protein in each sample to ensure equal protein loading. Sodium dodecyl sulfate-polyacrylamide gel electrophoresis was used to separate the proteins according to their molecular weight. Briefly, 15 µg of proteins (denatured) along with a molecular weight protein marker (Bio-Rad Kaleidoscope marker; Bio-Rad Laboratories) were loaded onto an acrylamide gel consisting of a 5% acrylamide stacking gel stacked on top of a 10% acrylamide resolving gel and run through the gel by application of 100 V for 1 h. Proteins were transferred from the gel to a polyvinylidene difluoride membrane using a wet-blotting method. The membrane was blocked with 5% Marvel in PBS containing 0.1% Tween20 (PBS-T), and probed with the following antibodies:

Rabbit anti-caspase 1 (Sigma Aldrich, cat. no. SAB5700660), rabbit anti-cleaved IL-1β (mature form) (Cell Signaling, cat. no. 83186), rat anti-IL-1β (pro-form) (ThermoFisher, cat. no. 17-7114-80), rabbit phospho-IκB (Ser 32, Cell Signaling, cat. no. 2859), total rabbit IκB (Cell Signaling, cat. no. 4812), rabbit NF-κB (Abcam, cat. no. ab16502), rabbit p-NF-κB (Abcam, cat. no. ab86299), mouse p-ERK1/2 (Sigma-Aldrich, cat. no. M-9692), rabbit ERK1/2 (Cell Signaling, cat. no. 4695) and β-actin (Sigma-Aldrich, cat. n. A1978). The enhanced chemiluminescence method of protein detection using enhanced chemiluminescence reagents (ECL Plus; Amersham GE Healthcare, Buckinghamshire, UK) was used to detect labeled proteins. Densitometry of films was performed using the Image J 1.42q software (available at http://rsb.info.nih.gov/ij/ (accessed on 24 October 2022), USA). Results of target protein expression are expressed as the ratio of non-phosphorylated form or densitometry of the endogenous controls β-actin.

### 4.7. CM-H2DCFDA Fluorescence Measurement of Reactive Oxygen Species

Chloromethyl 2′, 7′-dichlorodihydrofluorescein diacetate (CM-H2DCF-DA, Invitrogen, Paisley, UK) is a cell-permeable compound that following intracellular ester hydrolysis is oxidized to fluorescent 2′, 7′-dichlorofluorescein (DCF) by O_2_- and H_2_O_2_, and can therefore be used to monitor the intracellular generation of ROS [68]. In order to quantify ROS levels, differentiated U937 macrophages were washed twice with PBS and incubated for 30 min with 50 µM CM-H2DCF-DA diluted in Opti-MEM. Then, cells were again washed twice with PBS to remove the remaining CM-H2DCF-DA and stimulated with recombinant SARS-CoV-2 MOSAIC protein at different times as indicated. Tert-Butyl hydroperoxide at 0.77 mM was used as a positive control. Fluorescence intensity was analyzed by flow cytometry (FACSVerse; BD Biosciences, Madrid, Spain). A minimum of 10,000 cells per sample were analyzed with Flow-Jo standard software (TreeStar Inc., Ashland, OR, USA). Results were expressed as ROS fluorescence intensity which indicates DCF fluorescence in relative fluorescence units (RFU).

### 4.8. CM-FDA Fluorescence Measurement of Thiols

In order to quantify total intracellular thiol levels, differentiated U937 macrophages were stimulated with recombinant SARS-CoV-2 MOSAIC protein. At the end of the experiment, cells were collected, washed with PBS, and suspended in 12.5 µM 5-chloromethylfluorescein diacetate (5-CM-FDA) (Invitrogen, Paisley, UK) for 15 min. Then, cells were washed, and the fluorescence intensity was analyzed by flow cytometry (FACSVerse; BD Biosciences, Madrid, Spain). A minimum of 10,000 cells per sample were analyzed with Flow-Jo standard software (TreeStar Inc., Ashland, OR, USA). Results were expressed as fluorescence intensity which indicates CMFDA fluorescence in relative fluorescence units.

### 4.9. Measure of GSH

GSH-GloTM Glutathione Assay kit (Promega, cat. no. TB369, Madison, WI, USA) was used according to manufacturer instructions, which is a luminescence-based assay for the detection and quantification of glutathione. This assay is based on the conversion of a luciferin derivative to luciferin in the presence of glutathione catalyzed by glutathione-S-transferase (GST). 

### 4.10. Statistical Analysis 

Statistical analysis of results was carried out by non-parametric analysis. *p* < 0.05 was considered statistically significant. Data were displayed as medians and interquartile range values. When the comparisons concerned more than two groups, an analysis of variance (Kruskal-Wallis test) was first performed. In the case of a significant global difference, between-group comparisons were assessed by Dunn’s post hoc test, which generalizes the Bonferroni adjustment procedure. When the comparisons concerned only two groups, between-group differences were analyzed by the Mann-Whitney test. The correlation was analyzed by Spearman ρ non-parametric analysis.

## Figures and Tables

**Table 1:** Basal clinical data of patients. Data were acquired at the moment of tracheal biopsy. Data are *n* (%) or median, interquartile range (IQR) unless otherwise stated. 28-day mortality: death within 28 days of hospital admission; ALT: alanine aminotransferase; BMI: body mass index; COPD: chronic obstructive pulmonary disease; CRP: C reactive protein; LDH: lactate dehydrogenase; PAFiO2 = fraction of PaO2/FiO2; * *p* < 0.05 vs. control cohort; ^#^ *p* < 0.05 vs. B.1 variant.

Variable	Control Cohort	Covid-19 (B.1)	Covid-19 (B.1.1.7 VOC)
Number of patients	32	31	20
Age (years) [IQR]	65.2 [58.5–74]	67.7 [60.5–75.5]	80 [69.7–91.7] *^, #^
Male (%)	26 (81.2)	18 (58.1)	9 (45)
Exitus, *n* (yes %)	19 (59.4)	17 (54.8)	20 (100) *^, #^
28-day mortality, *n* (yes %)	13 (40.6)	4 (12.9)*	19 (95) *^, #^
Smoker (yes %)	17 (53.1)	11 (35.5)	8 (40)
Diabetes (yes %)	6 (18.7)	6 (19.3)	7 (35)
COPD (yes %)	5 (15.6)	0 (0)	2 (10)
Arterial hypertension (yes %)	15 (46.8)	14 (45.1)	17 (85) *^, #^
Dyslipemia (yes %)	11 (34.4)	11 (35.4)	11 (55)
BMI (kg/m2) [IQR]	26.8 [21.4–40.9]	32 [28.8–33.3]	30.2 [25.7–33.7]
Lymphocytes (10 * 9/L) [IQR]	0.95 [0.5–1.3]	1.05 [0.5–1.27]	0.67 [0.3–0.8]
Neutrophils (10 * 9/L) [IQR]	9.4 [6.3–12.8]	8.7 [5.9–11.2]	14.2 [6.5–20.5]
CRP (mg/L) [IQR]	8.4 [0.8–15.4]	5.8 [0.3–9.2]	14.05 [6.7–19.8] *^, #^
LDH (U/L) [IQR]	702 [630–774]	584.3 [496–666]	951 [692–1111] *^,#^
D-dimer (ng/mL) [IQR]	332 [263–425]	1593 [536–1929]	2797 [251–1473]
Procalcitonin (ng/mL) [IQR]	3.49 [0.3–1.9]	0.43 [0.07–1.25] *	7.45 [0.08–7.6]
pO2 (mmHg) [IQR]	148.1 [98.1–188.5]	138.1 [80.5–174.8]	92.2 [53–115] *, ^#^
pCO2 (mmHg) [IQR]	42.9 [37–52]	48.9 [42.2–52.7]	45.6 [41–52]
PaFiO2 [IQR]	279.8 [214–366]	240.1 [193–258]	220.2 [166–274]
HCO3 (mmoL/L) [IQR]	24.8 [20.4–27.9]	31.6 [27.8–33.6] *	27.9 [24.8–31.3]
Ferritin (µg/L) [IQR]	855 [240–1305]	930.9 [399–1443]	1045 [389–1448]
ALT (U/L) [IQR]	41.8 [10.7–60]	73.3 [32–104]	61.8 [23–70]
Systemic corticosteroids, *n* (%)	17 (53.1)	25 (80.6) *	18 (90) *

**Table 2:** NLRP3-inflammasome correlations with analytical parameters. ALT: alanine aminotransferase; CRP: C reactive protein; LDH: lactate dehydrogenase.* *p* < 0.05.

NRLP3 mRNA Expression vs.	Spearman ρ	*p* Value
Age (years)	0.4065	0.0092 *
Leukocytes (10^9^/L)	0.2122	0.194
Lymphocytes (10^9^/L)	−0.1716	0.2962
Neutrophils (10^9^/L)	0.2370	0.1464
Monocytes (10^9^/L)	0.04033	0.8074
CRP (mg/dL)	0.5374	0.0003 *
LDH (U/L)	0.5193	0.0016 *
Ferritin (µg/L)	0.3262	0.0597
ALT (U/L)	−0.04604	0.7867

## Data Availability

The data presented in this study are available on request from the corresponding author.
